# *Klebsiella pneumoniae* carbapenamases in *Escherichia coli* isolated from humans and livestock in rural south-western Uganda

**DOI:** 10.1371/journal.pone.0288243

**Published:** 2023-07-13

**Authors:** Barbra Tuhamize, Benon B. Asiimwe, Kennedy Kasaza, Wilber Sabiiti, Mathew Holden, Joel Bazira

**Affiliations:** 1 Department of Microbiology, Faculty of Medicine, Mbarara University of Science and Technology, Mbarara, Uganda; 2 Department of Medical Microbiology, School of Biomedical Sciences, College of Health Sciences, Makerere University, Kampala, Uganda; 3 Division of Infection and Global Health, School of Medicine, University of St Andrews, St Andrews, United Kingdom; Cornell University, UNITED STATES

## Abstract

**Background:**

The accumulation of resistance genes in *Escherichia coli* (*E*. *coli)* strains imposes limitations in the therapeutic options available for the treatment of infections caused by *E*.*coli*. Production of *Klebsiella pneumoniae* carbapenemase (KPC) by *E*. *coli* renders it resistant to broad-spectrum β-lactam antibiotics. Globally there is existing evidence of spread of carbapenem-resistant *E*. *coli* in both humans and livestock driven by acquisition of the several other carbapenemase genes. Overall, there is little information regarding the extent of KPC gene distribution in *E*. *coli*. We set out to determine the prevalence, and evaluate the phenotypic and genotypic patterns of KPC in *E*. *coli* isolated from humans and their livestock in rural south western Uganda.

**Methods:**

A laboratory-based, descriptive cross-sectional study was conducted involving 96 human and 96 livestock isolates collected from agro-pastoralist communities in Mbarara district in south western Uganda. Phenotypic and molecular methods (PCR) were used for presence and identification of KPC genes in the *E*. *coli* isolates. A chi-square test of independence was used to evaluate the differences in resistant patterns between carbapenems and isolates.

**Results:**

The overall prevalence of carbapenem resistance by disk diffusion susceptibility testing (DST) for both humans and livestock isolates were 41.7% (80/192). DST-based resistance was identical in both human and livestock isolates (41.7%). The prevalence of carbapenem resistance based on Modified Hodge Test (MHT) was 5% (2/40) and 10% (4/40) for humans and livestock isolates respectively. Both human and livestock isolates, 48.7% (95/192) had the KPC gene, higher than phenotypic expression; 41.7% (80/192). *bla*KPC gene prevalence was overall similar in human isolates (51%; 49/96) vs livestock isolates (47.9%; 46/96). Approximately, 19% (15/80) of the isolates were phenotypically resistant to carbapenems and over 70% (79/112) of the phenotypically sensitive strains harbored the *bla*KPC gene.

**Conclusion:**

Our results suggest that both human and livestock isolates of *E*. *coli* in our setting carry the *bla*KPC gene with a high percentage of strains not actively expressing the blaKPC gene. The finding of fewer isolates carrying the KPC gene than those phenotypically resistant to carbapenems suggests that other mechanisms are playing a role in this phenomenon, calling for further researcher into this phenomenon.

## Introduction

Carbapenem-resistant *Enterobacteriaceae* (CRE), are strains of gram-negative, rod shaped, facultative anaerobic bacteria that are resistant to carbapenems. carbapenems are a class of broad-spectrum antibiotics such as; imipenem, ertapenem and meropenem, resistant to hydrolysis by beta-lactamase currently used worldwide to treat bacteria resistant to other types of agents [1]. These pathogens cause both hospital and community acquired infections including, urinary tract infections (UTIs), bloodstream infections, hospital and healthcare-associated pneumonias, various intra-abdominal infections, peritonitis, meningitis, sepsis and medical device associated infections. *Escherichia Coli* is one of the most important pathogen for humans [2].

CRE have also been reported in food producing animals attributed to irrational use of antibiotics in animal husbandry practices [3]. Humans get exposed to the resistant strains harboring the encoding genes through ingestion of contaminated food or direct contact with animals, especially in regions where people frequently live in close contact with livestock animals [3].

In a systemic review of 68 articles, carbapenemase encoding genes, *bla*VIM, *bla*KPC, *bla*NDM, *bla*OXA and *bla*IMP were detected in companion animals and two studies found that 33% to 67% of exposed humans on poultry farms carried carbapenemase encoding genes related to isolates from the farm environment. In 27 studies that selectively screened for CRE, a prevalence of less than 1% among livestock and companion animals was found in Europe, 1% to 15% in Asia, and 2% to 26% in Africa [4].

Carbapenem-resistant *Enterobacteriaceae* are increasingly becoming a public health threat worldwide in hospital and community settings, posing grave implications in immune compromised hosts, with *E*. *coli* so far being the most important pathogen for humans. The potential bilateral dissemination of resistant strains or even their encoding determinants between humans and animals through direct contact or ingestion of contaminated food poses a worrisome public health risk, and calls for regular screening for presence of resistance genes as well as evaluation of expression of such genes.

*Klebsiella pneumoniae* carbapenemase (KPC) is one of the major causes of resistance to carbapenems in *Enterobacteriaceae* [5]. KPC enzymes include a major family of class A serine carbnapenemases, mainly produced by *K*. *pneumoniae* [6, 7]. KPC dissemination stresses the role of *bla*KPC gene in the spread of antimicrobial resistance. As such, strains harboring *bla*KPC gene are a major cause of concern for healthcare systems around the world [8].

There is paucity of information regarding the extent and/or resistant patterns of KPC given that previous studies have focused on genes like *blaO*XA, *bla*VIM and *bla*NDM in our setting [9]. Thus, this study aimed at establishing the phenotypic and genotypic prevalence of KPC in *E*. *coli* isolated from humans and corresponding livestock in rural south western Uganda.

## Methods

### Study design and setting

This was a laboratory-based, descriptive cross-sectional study in which we tested *E*. *coli* isolated from stool samples of humans and their corresponding livestock (either goats, chicken, cattle or swine) in the agro-pastoralist communities of Mbarara district South western Uganda, to evaluate the presence and expression of the *bla*KPC gene.

The study population comprised of isolates stored in Microbiology laboratory of Mbarara Regional Referral Hospital (MRRH). These isolates were previously collected from a One Health study Holistic Approach to Unravel Antimicrobial Resistance in East Africa (HATUA) conducted in this region between August 2018 and December 2020. In this study, informed consent was obtained verbally then urine samples were collected from farmers (referred to as humans elsewhere in the document) in different households of Bwizibwera and Rubaya and cultured overnight on Cysteine, lactose, and electrolyte-deficient agar (CLED) and Blood agar. Humans whose samples had colonies indicative of growth, were followed up in their communities and stool samples were taken from their livestock (sheep, goats, cattle, chicken and swine) if they had any. The livestock samples were cultured on Eosin Methylene Blue Agar (EMBA). Colonies indicative of growth in both human and livestock samples were identified by gram staining and further by biochemical tests. Pure colonies were appropriately labelled and stored in Tryptic soy Broth in the isolate bank in Microbiology laboratory.

### Laboratory procedures

#### *E*. *coli* Sub cultures and biochemical tests

A total of 96 archived human and 96 corresponding livestock *E*. *coli* isolates were selected from the microbiology isolate bank and a loopful streaked onto MacConkey with crystal violet agar and incubated for 18–24 hours, at 37°C to obtain pure growth. Gram negative bacilli colonies which were citrate negative, indole positive, urea negative and motile were identified as *E*. *coli*.

### Disk diffusion susceptibility testing

DST was performed using the Kirby Bauer disk diffusion method on Mueller Hinton Agar (MHA) plates in accordance with the clinical laboratory standards institute (CSLI) [10]. Using a sterile wire loop, *E*. *coli* colonies (isolates and controls: *E*. *coli* BAA 1706 *bla*KPC positive and BAA 1705 *bla*KPC negative) were emulsified into sterile saline (0.9%) and the turbidity of the suspension adjusted to 0.5 McFarland standard. Using a sterile swap, the emulsified colonies were introduced onto an MHA plates by surface spreading. The antimicrobial disks imipenem (IMP, 10μg) and meropenem (MER, 10μg) were introduced onto the MHA plates and cultured overnight. Resistance to the carbapenems was determined by reading zonal diameters basing on CLSI guidelines 2019 (≤22mm = Resistant; R,> 22 = Sensitive; S).

### Phenotypic screening for carbapenem resistance in *E*. *coli*

The modified Hodge Test (MHT) was used to determine if resistance to carbapenems was caused by carbnapenemases. A Muller Hinton agar plate was inoculated with a susceptible *bla*KPC control *E*. *coli* BAA 1705 strain (McFarland 0.5, diluted 1/10) as for disk diffusion, one ertapenem Neo-Sensitabs (ERT, 10μg) and one meropenem Neo-Sensitabs (MER, 10μg) were applied onto the plate approximately 30 mm apart from each other. A suspension of the microorganism to be tested for carbapenemase was adjusted to McFarland 0.5 standard and a loop was used to make a streak passing through the two carbapenem disks. Two more streaks were placed perpendicularly making a cross and the plates incubated for 18–24 hours at 35–37°C. Alteration in the shape (indentation) of the zones of inhibition around the test organism was considered indicative of the presence of a carbapenemase.

### Genotypic detection of *bla*KPC carbapenemase encoding genes

Bacterial genomic DNA from the isolates, the positive and negative *bla*KPC control strains (*E*. *coli* BAA 1706 & BAA 1705 respectively) was extracted using the Zymo-Research quick-*g*-DNA Miniprep kit (INQABA, South Africa) and manufacturer’s instructions was followed.

The presence of *bla*KPC was established by PCR amplification using a method described earlier by Shanmugam, Meenakshisundaram [11] with the following primer sets. Forward Primer: 5’-GCTCAGGCGCAACTGTAAG-3’, Reverse Primer: 5’-AGCACAGCGGCAGCAAGAAAG-3’. The PCR master mix was prepared as follows: 12.5 μL master mix consisting of one *Taq* quick-load 2x master mix containing standard buffer, dNTPs & Taq polymerase (M0486S); 1.0 μL forward (75 μM), 1.0 μL reverse (75 μM), 5 μL DNA template and 5μL RNAase-Free-H_2_O making up to 25μL final reaction volume.

The PCR amplification was carried out in a conventional PCR Thermocycler (Multigene Optimax,), with the following cycling temperature profiles: Initial denaturation of 95°C for 30 s, followed by DNA amplification stage with 30 cycles (95°C for 30 s, 55°C for 45 s, and 68°C for 1 min) and final extension cycle of 68°C for 5 min.

DNA Amplicon was electrophoresed using 1.5% agarose gel, in 1x Tris-Borate EDTA buffer (TBE), 5μL Safe View Classic^TM^ DNA stain (cat # G108), 6x loading dye (Thermo Scientific #R0611), and DNA ladder/marker 100 bp (NEB-Biolabs #N3231L). Electrophoresis was run at 100V and 80mA for 1 hour. Bands were visualized using the Gene-Flash Trans-illuminator [11].

### Data analysis

Data collected were entered in Microsoft Excel. Then imported for analysis in Statistical Package for the Social Sciences, SPSS version 20, SPSS Inc. Data was presented in frequencies and proportions, and presented in tables and histograms. Pearson’s *Chi* square was used to establish associations in bivariate analyses. For all tests, a *P ≤ 0*.*05* was considered significant.

### Ethical considerations

The study was approved by Institutional Ethical Review Committee of Mbarara University of Science and Technology (Study number MUREC1/7 14/01-20), and permission was granted by the ongoing studies’ principal investigators.

## Results

A total of 192 *E*. *coli* confirmed isolates (96 humans and 96 corresponding livestock isolates were tested for resistance to carbapenems using Kirby Bauer disk diffusion method. The phenotypically resistant isolates (n = 80) were screened for carbapenemase production using MHT. Genotypic screening for KPC gene was done for all isolates (N = 192).

### Isolate characteristics and an overview of the resistance patterns to carbapenems

A total of 192 isolates (96 human and 96 corresponding livestock) were collected from 96 households. Our study found that the phenotypic resistance by DST was similar for human vs livestock isolates as 41.7% (total 80; 40 human and 40 corresponding livestock) out of the 192 isolates. Of the 80 phenotypically resistant (by DST) isolates, only a small proportion (6/80) were phenotypical resistant by MHT test. Nearly a half of the total isolates (49.5%, 95/192) were found to harbor the *bla*KPC with the *bla*KPC prevalence in human isolates slightly higher than that of livestock isolates “Table 1”. In addition, 32 households had both human and livestock isolates having *E*. *coli* harboring the KPC gene while 29 households had either human or livestock isolate positive for *bla*KPC gene “Table 1”.

**Table 1:** Isolate characteristics and overview of the resistance patterns to carbapenems.

**Laboratory Test**	**Isolate category, n (%)**		**Total, n (%)**
	**Human**	**Livestock**	
DST (N = 192)	40 (41.7%)	40 (41.7%)	80 (41.7%)
MHT (N = 80)	2 (5%)*	4 (10%)*	6 (7.5%)
PCR (N = 192)	49 (51%)	46 (47.9%)	95 (49.5%)

**DST:** Disk diffusion susceptibility testing, **MHT**: Modified Hodge Test, **PCR**: Polymerase Chain Reaction.

^a^ Number of resistant isolates to carbapenems (by DST and MHT) and number of isolates harboring *bla*KPC gene (PCR).

* Number of resistant isolates by MHT from the DST-resistant isolates under each isolate category.

### Comparisons of prevalence amongst humans and livestock based on DST

The prevalence of individual drugs was as follows: resistance in humans was 31.3% (30/96) for meropenem and that of imipenem was 37.5% (36/96), and in livestock meropenem resistance was 34.4% (33/96) and 37.5% (36/96) for imipenem. There was no difference in the resistance patterns of individual drugs in humans and livestock: meropenem (*p =* 0.759) and imipenem (*p =* 1.000). In addition, there was no significant difference observed in carbapenem resistance of individual drugs in human isolates (*p =* 0.447) and livestock isolates (*p =* 0.764).

### Comparison of phenotypic and genotypic resistance patterns among *E*. *coli* isolates

**Fig 1:**
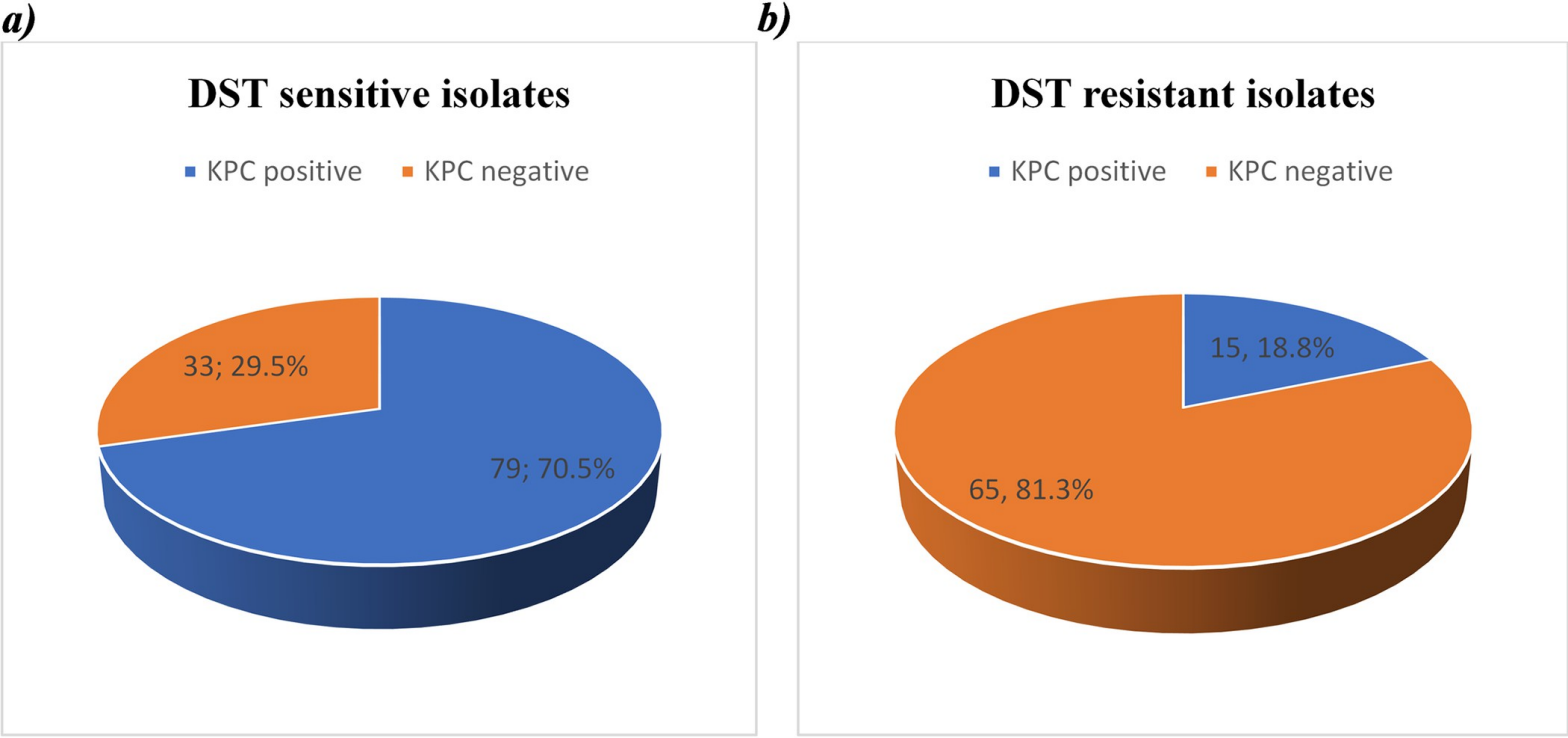
Pie charts showing the proportion of KPC positive *E*. *coli* isolates from both sensitive and resistant strains by DST. As shown in “Fig 1”, 70.5% of isolates initially susceptible with DST were positive for the KPC gene, conversely only 18.8% DST phenotypic carbapenem-resistant *E*. *coli* had KPC gene identified.

Overall, the prevalence of *bla*KPC gene in *E*. *coli* isolates studied was higher 49.5% (95/192) compared to that based on phenotypic expression, that is 41.7% (80/192). 15 out of the 80 (18.8%) isolates that were resistant to carbapenems had *bla*KPC gene. In addition, the DST phenotypically susceptible strains that harbored the *bla*KPC gene were 70.5% (79/112), indicative of a higher gene pool for *bla*KPC gene “Fig 1”. Out of the 6 MHT phenotypic resistant isolates, 1 harbored *bla*KPC gene.

## Discussion

*Escherichia coli* is one of the major etiologic agents causing a variety of infections in humans. Carbapenem-resistant *E*. *coli* is increasingly becoming a public health concern worldwide. There is existing evidence of spread of carbapenem-resistant *E*. *coli* in both humans and livestock [12, 13]. In the present study we found that 80 out of 192 (41.7%) isolates were resistant to carbapenems by disk susceptibility testing method. Of these, 40 were humans and 40 were livestock isolates. The similarity in the proportion is likely due to the cross transmission of carbapenem-resistant *E*.*coli* between humans and livestock via consumption of water, direct contact with livestock animals [12] or via the food chain [14]. A lower prevalence in human isolates of 7% was observed in a study in Kenya. This could be due to a few number (40) of hospital isolates [15] different from our community-acquired human isolates and the sampling technique which involved wounds, sites of potential infections with other organisms other than *E*. *coli*.

The potential bilateral distribution of resistant strains or even their encoding determinants between humans and animals is a public health risk. A lower prevalence of 5% was obtained for carbapenem resistance in livestock (cattle) in a study conducted in the pastoralist communities in Kasese district in southwestern Uganda [16]. This could be due to difference in sampling since samples were only picked from cattle, while our study involved more than one kind of livestock, increasing the chance of obtaining samples with *E*. *coli*.

Our current study revealed a prevalence of the carbapenemase phenotype of 5% in humans and 10% in livestock among *E*. *coli* resistant to carbapenems. This is comparable to an earlier study of epidemiology of carbapenem-resistant *E*. *coli* which found a prevalence of 4.92% in hospital isolates studying resistance to imipenem [17], though our study used a more sensitive MHT and community-acquired isolates. In another study in Uganda the prevalence of MHT-based screening was high at 22.4%. The difference in the findings could be because, in this study, many organisms were studied and multiple methods with better sensitivities were used to study phenotypic expression [18]. Lower prevalence is seen in studies in hospital isolates from China (1.0%) [19], Germany (0.3%) [20] and surveillance study in Spain (0.04%) despite having larger sample sizes. The differences may be due to the liberal nature of acquiring drugs from pharmacies over-the-counter even without a prescription in Uganda [21], vs the restricted use of antibiotics in developed countries. Overall, in our study the MHT values are very low compared to those using DST. This difference may be due to the fact that MHT has reduced sensitivity in isolates showing weak positive results [22, 23]. In addition, DST could identify resistant isolates to carbapenems in the study, MHT narrows to a few resistance genes including *bla*KPC gene giving a lower prevalence if the latter method is used for identification. Equally, MHT is no longer recommended for detection carbapenamases due to the low specificity for detection of KPC [24].

In our study, an overall prevalence of *E*. *coli* with *bla*KPC gene (49.5%) was obtained. A high prevalence of *bla*KPC gene in human *E*.*coli* isolates (51%) obtained in our study was way high compared to the two previous studies that did not find any *bla*KPC gene in the carbapenem resistant *Enterobacteriaceae* isolates that were studied [17, 25]. Overall, there has been a rapidly increasing prevalence of carbapenem-resistant *Enterobacteriaceae* reported over the past decade which has increased concern in healthcare facilities and public health communities worldwide [26]. Furthermore, in livestock carbapenemase producing *Enterobacteriaceae* have been isolated with no evidence of *bla*KPC gene production in *E*. *coli* [27–29]. In a recent study by Iramiot and colleagues, 2020 on antimicrobial resistance at the human–animal interface in the pastoralist communities of Kasese District, no information regarding genetic epidemiology of carbapenamase producing *Enterobacteriaceae* was reported [12]. In another study on the phenotypic and genotypic analyses of antimicrobial resistance in livestock in Uganda by Okubo and colleagues, they reported about other genes responsible for carbapenamase resistance (*bla*ACT) [13], but not *bla*KPC gene. This suggests that the bacterial isolates might not have harbored the *bla*KPC gene then, and that in the current study, the *E*. *coli* might have acquired the gene from *Klebsiella*. *bla*KPC gene has been reported to be increasing in *Enterobacteriaceae* as plasmids are acquired from K. pneumoniae, particularly in *E*. *coli* elsewhere in clinical isolates [30–32].

It was observed in our study that the prevalence of carbapenem-resistant *E*. *coli* by DST method were 80 out of 192 (41.7%) and by PCR (for presence of *bla*KPC gene) were 95 out of 192 (48.7%). This indicates a high gene pool for *bla*KPC gene with cases of silent genes in some of the samples. The existence of silent genes could be rather be explained by the low drug selective pressure as a result of lower overall usage of carbapenems [33, 34] in the community setting. More evidence is shown by the large proportion; 79 out of 112 (70.5%) of *bla*KPC positive, but DST-sensitive *E*. *coli* observed in our study. Similar findings relating to silent genes have been also documented elsewhere [11, 35]. Conversely, only 15 out of 80 (15.8%) DST-based carbapenem-resistant *E*. *coli* had *bla*KPC gene identified. This is due to the fact that the *E*. *coli* isolates could have carried other genes (like *bla*OXA, *bla*VIM, *bla*NDM) responsible for carbapenem resistance other than *bla*KPC as observed in previous studies. The study was however limited by a small sample size thus, these findings might not be used for generalization.

## Conclusions

Our results confirm the presence of carbapenem‐resistant *E*. *coli* among human and livestock in this rural South western Uganda and are likely to increase as carbapenems are increasingly being used for treatment of human infections.

## Recommendations

We recommend continued surveillance of carbapenem resistance coupled with cautious prescription of carbapenems.
